# The time is ripe to investigate human centromeres by long-read sequencing^†^

**DOI:** 10.1093/dnares/dsab021

**Published:** 2021-10-05

**Authors:** Yuta Suzuki, Shinichi Morishita

**Affiliations:** Department of Computational Biology and Medical Sciences, Graduate School of Frontier Sciences, The University of Tokyo, Kashiwa, Chiba 277-8568, Japan

**Keywords:** long-read sequencing, centromere, genome assembly, haplotyping, CpG methylation

## Abstract

The complete sequencing of human centromeres, which are filled with highly repetitive elements, has long been challenging. In human centromeres, α-satellite monomers of about 171 bp in length are the basic repeating units, but α-satellite monomers constitute the higher-order repeat (HOR) units, and thousands of copies of highly homologous HOR units form large arrays, which have hampered sequence assembly of human centromeres. Because most HOR unit occurrences are covered by long reads of about 10 kb, the recent availability of much longer reads is expected to enable observation of individual HOR occurrences in terms of their single-nucleotide or structural variants. The time has come to examine the complete sequence of human centromeres.

## 1. Background

Centromeres have been one of the most mysterious parts of the human genome since they were characterized, in the 1970s, as large tracts of 171-base pair (bp) strings called alpha-satellite monomers.^1^^,^^2^ With a growing body of evidence suggesting their relevance to human diseases as sources of genomic instability or as repositories of haplotypes containing causative mutations,^3–8^ it has become more important to investigate the underlying sequence variations in centromeric regions.^9^^,^^10^

**Figure 1:**
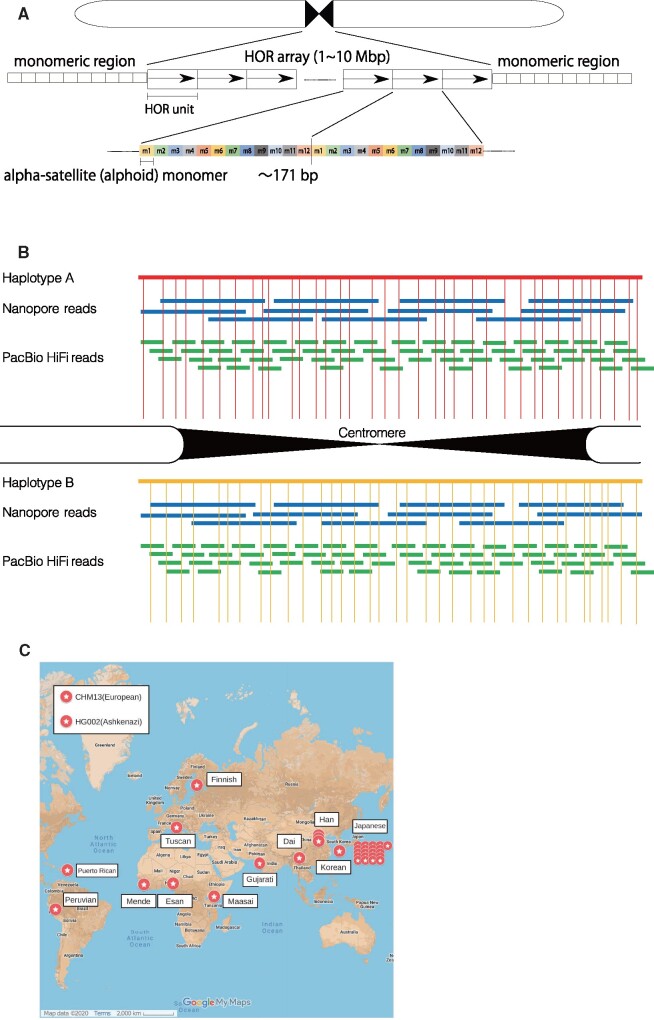
(A) Schematic of a typical DNA sequence structure of human centromeric regions. The entire region consists mostly of alphoid monomers of 171 bp. The core centromeric regions (up to several million base pairs) with an HOR structure are sandwiched by the pericentromeric (monomeric) regions, where monomers are arranged tandemly without an HOR. (B) A special strategy of sequencing human centromeres using Nanopore ultralong reads, PacBio HiFi reads, and other long-read data. The vertical lines represent positional markers to overlap long reads unambiguously. We need to collect two different long-read datasets for sequencing two individual haplotypes separately, a non-trivial task because two haplotypes are repetitive and difficult to distinguish. Thus, the telomere-to-telomere project used a haploid genome, CHM13, to reconstruct a complete genome. (C) Geographic locations of the 36 samples analysed in this study.^44^ CHM13 and HG002 are placed in the upper left corner because it is hard to tell which region they are from.

Human centromeric regions have nested repeat structures. Namely, a series of distinctively divergent alpha-satellite monomers compose a larger unit called a higher-order repeat (HOR) unit, and copies of an HOR unit are tandemly arranged thousands of times to form large, homogeneous HOR arrays. While HOR units are chromosome specific and consist of 2–34 alpha-satellite monomers, copies of an HOR unit are almost identical (95–100%) within a chromosome (Fig. 1A).^11–17^

The total HOR array length of each chromosome differs markedly among individuals^7^^,^^18^ and human populations.^19–21^ Structural alterations such as unequal crossing-over and/or gene conversion are thought to be among the major driving forces of this centromeric variation.^22^^,^^23^ Other types of variation occur within HOR arrays, such as single-nucleotide variations (SNVs) between paralogous HOR units^21^^,^^24^^,^^25^ and structurally variant HORs, which consist of different numbers and/or types of alpha-satellite monomers.^21^^,^^26–28^ However, the importance of structurally variant HORs is unknown because they are difficult to detect comprehensively via traditional approaches such as restriction enzymes sensitive to alpha-satellite monomers, Southern blotting, or the analysis of *k*-mers unique to centromeric regions in short reads obtained in the 1000 Genomes Project.^29^

Recently, the advent of long-read sequencing technologies has paved the way for direct, comprehensive observation of sequence variations among human populations.^30–34^ Long-read sequencing is capable of yielding contiguous reference sequences of centromeres for several species,^35^^,^^36^ and reconstruction of whole centromeric sequences for a human haploid genome is now possible despite their idiosyncratic repeat structures.^37–40^

## 2. Complete sequences of human centromeres

Long-read sequencing has yielded contiguous sequences of centromeres in several species.^35^^,^^36^ Recently, a number of whole centromeric arrays reconstructed with ultralong nanopore reads and/or accurate PacBio HiFi reads have been reported for a haploid genome, such as complete hydatidiform mole CHM13, which avoids the difficulty of distinguishing between two very similar haplotypes in a diploid genome (Fig. 1B).^37–40^ As of 2021, the best method for reconstructing human centromeres would be to combine a variety of long-read data, such as Nanopore ultralong reads of length >100 kb, PacBio ∼20 kb HiFi reads of base accuracy ∼99.9%, Illumina linked short reads, Bionano optical mapping data, and Hi-C data (Fig. 1B). To reconstruct human centromeres from these reads, one might consider the use of genome assemblers for processing long reads, such as HiCanu^40^ and hifiasm.^41^ Although these assemblers can assemble non-repetitive regions accurately, they are not designed to handle highly repetitive regions such as human centromeres.

The telomere-to-telomere project (T2T)^38^ has used a special approach tailored to centromeric repeats so as to generate a minimum tiling path, a series of overlapping Nanopore ultralong reads, that span the centromere of a focal chromosome.^38^^,^^42^ To determine overlapping Nanopore ultralong reads accurately, the T2T project first built a catalog of structural and single-nucleotide variants in ∼2 kb canonical HORs in the case of chromosome X and used them as positional markers to overlap ultralong reads. Afterwards, the T2T project aligned PacBio HiFi reads and Illumina short reads to the tiling path unambiguously and polished the unique positions in the assembly using Illumina short reads. The T2T project manually corrected regions that were structurally inconsistent with Bionano optical map data. The base accuracy was estimated to be 99.991% based on X-specific BACs.

## 3. Unassembled long reads are useful for investigating HOR variants in diploid human genomes

While reference-quality *de novo* assembly of human centromeres requires a large amount of long reads, which is quite costly and remains a demanding task involving substantial manual curation, the use of unassembled long reads is less costly and has promise for investigating HOR variations within centromeric regions of diploid genomes in a cost-effective manner.^43^ Furthermore, observing HOR variants in unassembled long-read sequences allows us to estimate the frequency distribution of HOR variants and to analyse major and minor HOR components in diploid human centromeres.

To investigate inter-individual variation within the centromeric array, we analysed publicly available, single-molecule, real-time sequencing (PacBio) reads collected from 12 samples from geographically diverse origins—three from Africa (Mende, Sierra Leone; Esan, Nigeria; and Maasai, Kenya), two from Europe (Toscani, Italy, and Finland), five from Asia (Gujarati, India; Dai, China; and three from Han, China), and two from Latin America (Puerto Rico and Peru). We also analysed 21 newly sequenced Japanese datasets and three previously described samples: AK1 (Korean), HG002 (Ashkenazi), and CHM13 (European)^31^^,^^32^^,^^34^ (Fig. 1C). Thus, we analysed a total of 36 samples.^44^

## 4. Prevalence of non-canonical variant HORs

We detected variant HORs that were diverse in terms of presence and abundance among the samples. In chromosome X, the canonical HOR consists of 12 monomers; this was the most frequent pattern found in reads across all of the datasets (96.2–98.4% of all HOR types). In addition to the canonical 12-mer HOR, 51 variant HORs were defined, ranging in size from 2- to 23-mers. While some variant HORs were shared by all 36 samples, others were specific to or missing from a few samples.

**Figure 2:**
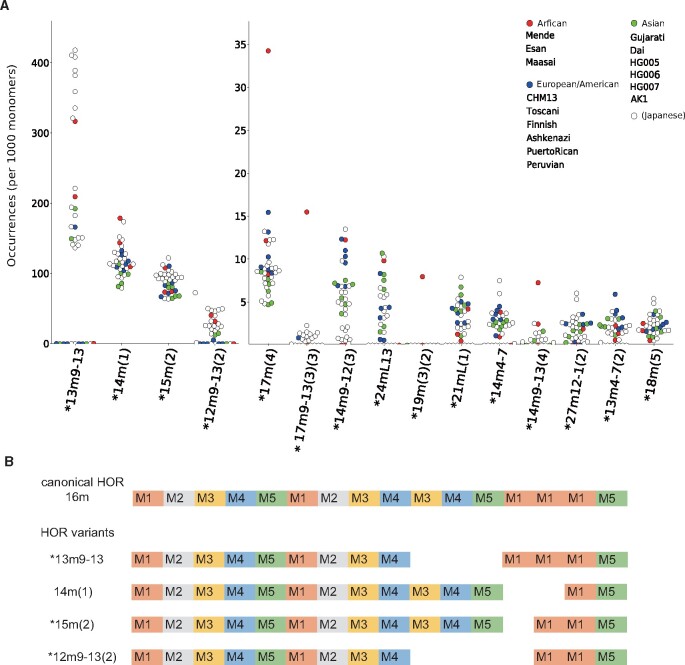
Detected HORs in chromosome 17 and their relative frequencies among 36 samples. (A) Relative frequencies (per 1,000 monomers) of detected variant HORs for 36 samples in chromosome 17. Red, blue, green, and white circles represent Africa, Europe, Asia, and Japan, respectively. For example, the leftmost column shows the frequency distribution of HOR variant named 13m9-13. This HOR variant is observed 130–420 times per 1,000 monomers among any of African, European, Asian, and Japanese samples. (B) Previse structures of HORs are presented in rows. Rectangles indicate alphoid monomers and their classes. No gap is allowed between two adjacent monomers to be detected as a variant HOR unit. Five basic monomers are labelled with M1, M2, M3, M4, and M5 and are coloured orange, grey, yellow, blue, and green, respectively. The most abundant HOR at the top has 16 monomers and is hence labelled with ‘16m’. The other four HOR variants in rows are aligned with 16m and are labelled with their identifiers. For example, the structure 13m9-13 at the second row has 13 monomers but miss three after the 9th and before 13th monomers.

For chromosome 17, 91 distinct variants were detected, ranging in size from 5- to 39-mers. Notably, a 13-mer variant (13m9-13; the 10th, 11th, and 12th monomers had been deleted from the canonical 16-mer) was present at high frequency in approximately half of the samples, whereas it was generally missing from another half of samples (Fig. 2). Samples with the characteristic 13-mer variant exhibited the so-called Haplotype II, which has an estimated allele frequency of ∼35% in European populations.^25^^,^^45^ Prevalent variant HORs were also observed, including a 15-mer [15m(2)] and a 14-mer [14m(1)], suggesting that the canonical 16-mer was less stable than canonical HORs in chromosomes X or 11. Consequently, unlike chromosome X, the relative frequencies of canonical 16-mer HORs were highly divergent among the samples, ranging from 21.6 to 76.0%. The distribution of the remaining variant HORs across the individual samples was also markedly non-uniform.

## 5. Rapid evolution of variant HORs

To evaluate the diversity of variant HORs within a population, we quantitatively measured variation among the 21 Japanese samples. The SD of the variant HOR frequency was 45.05 events per megabase (Mb), which approximated the expected density of distinct variant HORs harboured by each individual genome. We next compared our results with a recent estimate of genome-wide structural variation (SV) detection from accurate circular-consensus long reads, which obtained a reliable set of ∼30,000 SVs for an individual genome, with respect to a reference genome.^34^ The average density of SVs for each of the 23 chromosomes (autosomes and X) was 21.16 SVs/Mb (SE* *=* *4.45 SVs/Mb); a two-tailed one-sample *t*-test confirmed that SVs were significantly more abundant in centromeric than in non-centromeric regions (*P *=* *6.51 × 10^−18^). Therefore, the centromeric array appears to change rapidly in terms of variant HORs.

Although canonical HOR patterns were detected in all samples, non-canonical variant HORs were more dynamic overall, because non-canonical HORs were likely to be specific to subsets of individuals across different populations or exhibit divergent frequencies even within a population, showing rapid evolution in the human centromeric arrays. It was technically crucial to enumerate variant HORs in long reads collected from individuals, but we do not have space in this review to explain how to do so (see the details in Ref. 44). For understanding the association between non-canonical HOR variants and focal diseases, population-wide profiling of variant HORs in case and control samples would be informative. If specific variants have functional implications, they could be useful as biomarkers.

## 6. Haplotype-specific evolution of the centromeric array

Several mechanisms contribute to the structural diversity within centromeric sequences: unequal crossover between sister chromatids, meiotic unequal crossover, gene conversion, and homologous recombination resulting in non-crossover products, to name a few. Among them, meiotic crossovers might arguably be excluded as a major driving force because they are suppressed near centromeric regions^7^^,^^46^ and, consequently, centromeric regions are reported to form large conserved linkage-disequilibrium blocks,^10^ as reconfirmed by an analysis of HOR variants observed in long reads.

**Figure 3:**
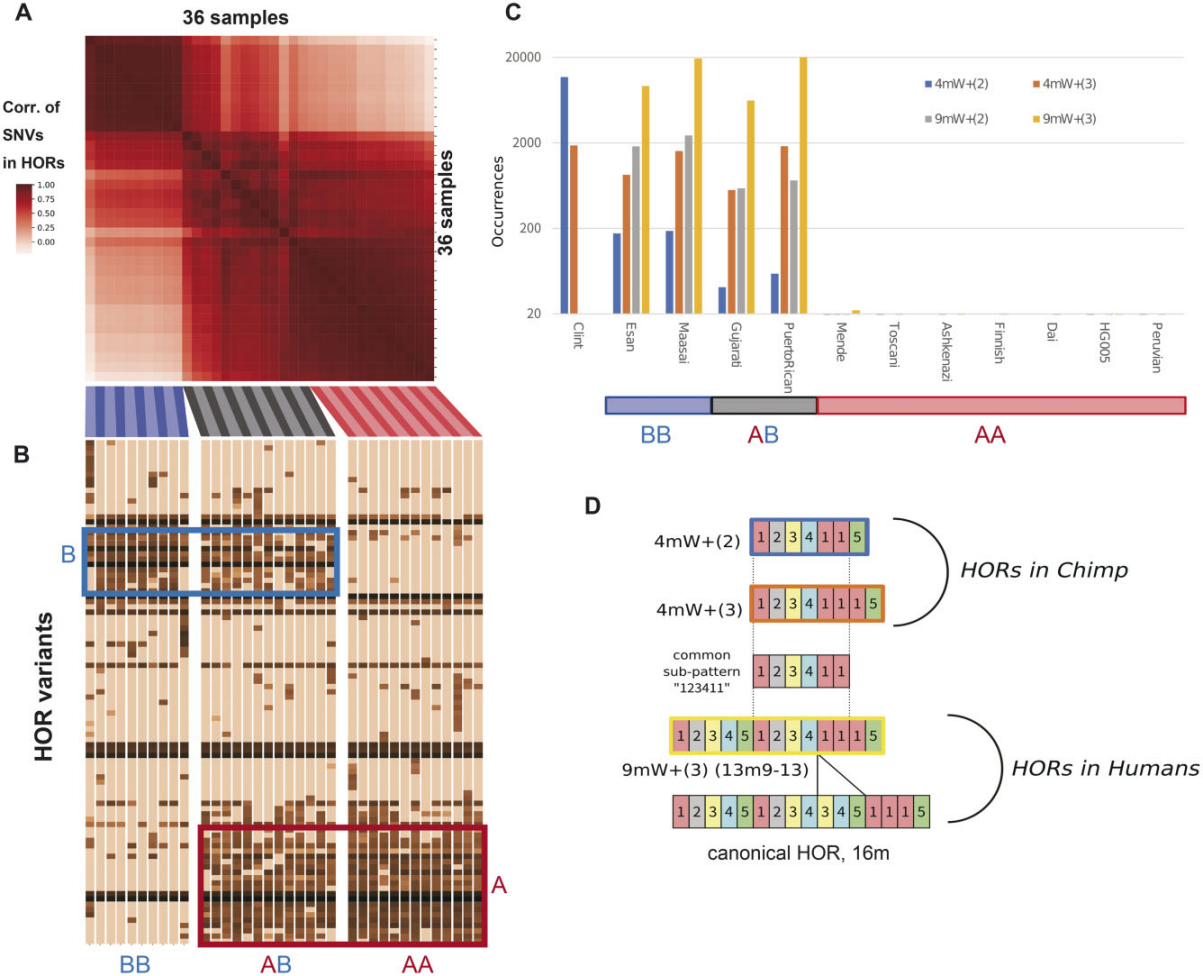
Haplotype-specific evolution of chromosome 17 centromeric arrays among 36 samples. (A) Correlation of SNV frequencies among 36 samples on the canonical 16-mer HOR units for chromosome 17. The strength of the correlation is indicated by the brightness of the colours shown in the left bar. Sample labels are coloured blue (BB), black (AB), or red (AA) according to the haplotype combination inferred by analysis of HOR variants. (B) Occurrence of variant HORs in each sample as a fingerprint of the haplotype. SVs were clustered by co-occurrence over the samples. A- and B-specific variant HORs are labelled in red and blue, respectively. Darker cells indicate that they are observed with higher frequency. Sample labels are coloured according to the haplotype combination (blue, BB; black, AB; red, AA). Observe a clear correspondence between the correlation of HOR SNV in Figure A and the clustering of HOR variants in (B). (C) Frequencies (in log scale) of B-specific variant HORs (in terms of generic monomers) in chimpanzee (Clint, leftmost) and human samples, Esan (second column), Maasai (third), Gujarati (fourth), and PuertoRican (fifth). (D) Schematic representations of HORs with the B-specific pattern. The numbered blocks represent the alphoid monomers (of supra-chromosomal family 3), which constitute the HOR patterns in human and chimpanzee. To represent five monomers (M1, M2, M3, M4, and M5), we here use the same colour coding as that in Figure 2B. This analysis revealed that a common pattern, 123411, with six monomers has evolved differently in the chimpanzee and human lineages.

For chromosome 17, the correlation of SNV frequencies was considerably diverse, depending on the pair of samples (Fig. 3A). Samples with highly correlated SNV frequencies often shared a similar set of HOR variants (Fig. 3B). For example, 10 samples that were coloured blue and labelled with BB (Maasai, Esan, and eight Japanese) were strongly correlated in terms of SNV frequencies; they also shared a characteristic pattern of variant HORs, such as the presence of HOR variants in the blue box or the absence of those in the red box. Another 13 samples that were coloured red and labelled with AA (Mende, Toscani, CHM13, Ashkenazi, Finnish, Dai Chinese, Han Chinese trio, Peruvian, and three Japanese) with shared SNVs exhibited the reverse pattern in terms of variant HORs. The variant 9mW+(3) in Fig. 3C and D, which is equal to 13m9-13 in Fig. 2, is a marker for an alternative allele (Haplotype II) for the chromosome 17 centromere in contrast to the wild-type allele (Haplotype I).^25^^,^^45^ Below, we refer to Haplotypes I and II as Haplotypes A and B, respectively, just for better readability. Our analysis indicated that many other variant HORs exhibited positive or negative correlations with the marker variant 9mW+(3) (13m9-13). The haplotype combination in each sample (AA, BB, or AB) was also evident in the pairwise correlation of SNV frequencies (Fig. 3A and B).

## 7. Distribution of Haplotype B-specific patterns in a chimpanzee centromeric array

To determine which haplotype, A or B, was ancestral in terms of centromere sequence evolution, we performed an HOR analysis using a chimpanzee (Clint) as the outgroup.^47^ Although chimpanzee centromeric arrays share some HOR structures with humans, we did not rely on existing information on HOR patterns.^16^ We used a set of 10 generic monomers, including five monomers (W1–W5) of supra-chromosomal family 3, to capture HOR patterns present in both chimpanzee and human.

Using the generic monomers, we identified HOR patterns that were shared by the human samples with Haplotype B (homozygous or heterozygous) but were absent from those homozygous for Haplotype A (Fig. 3C). These characteristic patterns shared an HOR sub-pattern (123411), which served as a Haplotype B-specific marker. Notably, this pattern was frequently observed in chimpanzee, although the contexts in which the breakpoints occurred differed slightly in human and chimpanzee (Fig. 3D). These findings implied that the pattern found in Haplotype B was originally shared by both species, but they might have evolved into distinct HOR arrays in each species. Subsequently, Haplotype A (in which the pattern was lost) had spread within the human population.

## 8. CpG methylation in human centromeres

CpG methylation has been linked to a variety of key biological phenomena including repression of gene transcription, repression of transposable elements, ageing, genomic imprinting, X-chromosome inactivation, and carcinogenesis. Short-read bisulphite sequencing has been a standard approach to evaluate CpG methylation; however, it has limitations in detecting CpG methylation in highly repetitive genomic regions (including centromeric regions) because it is extremely difficult to map short reads to repetitive regions. Fortunately, this problem can be solved by long-read sequencing, which allows direct observation of CpG methylation.

For example, PacBio single-molecule real-time sequencing monitors the process by which single deoxyribonucleotide is taken up by DNA polymerase, and it takes longer to monitor template DNA with methylated CpGs than template DNA with unmethylated CpGs.^48^ This property enabled the design of an observation method for CpG methylation, which was used to detect hypo-methylated regions within the centromere of an inbred strain of medaka fish.^36^

The electrical signal of nanopore sequencing is also sensitive to methylated CpGs.^49^ The T2T project used this method to reveal the landscape of CpG methylation in the chromosome X centromere and observed hypo-methylation across the pseudo-autosomal regions, in agreement with previous reports.^38^ They employed the same approach to analyse the chromosome 8 centromere and its CpG methylation organization, and located the hypomethylated region that was enriched in the centromeric histone CENP-A, suggesting the kinetochore binding site.^50^

Even with the latest technology, it is not yet possible to achieve a full assembly scheme to completely determine the centromeric repeats of a diploid genome. However, unassembled HOR variants and their CpG methylation status collected from a number of individuals are very useful to know what specific sequences of HOR variants are involved in the unmethylation of centromeric repeats.

Overall, long-read sequencing will enhance our understanding of the roles of CpG methylation in the human centromere.

## 9. Future perspectives

The technical feasibility of fully sequencing human centromeres using Nanopore’s ultralong reads and PacBio’s highly accurate HiFi reads is beginning to be understood. Two major approaches to understanding the human centromere were presented. One is the telomere-to-telomere project in the United States that aims to completely sequence centromeres using a haploid genome (hydatidiform mole CHM13). This would require a large collection of long leads, which is very costly as of 2021. On the other hand, we explored an alternative approach using a smaller amount of unassembled long reads to study HOR variants in the centromeres of diploid human genomes. Using this method, we have detected a variety of non-canonical HOR variants, which may correlate with large conserved linkage-disequilibrium blocks. To understand this fully, the complete sequences of centromeres for individuals are needed. Because the use of unassembled reads is less costly than the fully assembled approach, it is more feasible to perform population-scale analyses of the association of non-canonical HOR variants with diseases, such as those for which genome-wide association studies have indicated the presence of significantly associated SNPs around centromeres. In any case, the trade-off between sequencing cost and sample size needs to be considered to develop an optimal research plan.
